# The Role of Flavonoids on Oxidative Stress in Epilepsy

**DOI:** 10.1155/2015/171756

**Published:** 2015-01-11

**Authors:** Tâmara Coimbra Diniz, Juliane Cabral Silva, Sarah Raquel Gomes de Lima-Saraiva, Fernanda Pires Rodrigues de Almeida Ribeiro, Alessandra Gomes Marques Pacheco, Rivelilson Mendes de Freitas, Lucindo José Quintans-Júnior, Jullyana de Souza Siqueira Quintans, Rosemairy Luciane Mendes, Jackson Roberto Guedes da Silva Almeida

**Affiliations:** ^1^Postgraduate Program in Biotechnology, State University of Feira de Santana, 44036-900 Feira de Santana, BA, Brazil; ^2^Department of Physiology, Federal University of Sergipe, 49100-000 São Cristóvão, SE, Brazil; ^3^Federal University of Pernambuco, 50740-521 Recife, PE, Brazil; ^4^Federal University of Piauí, 64049-550 Teresina, PI, Brazil; ^5^Federal University of San Francisco Valley, 56304-205 Petrolina, PE, Brazil

## Abstract

*Backgrounds*. Oxidative stress can result from excessive free-radical production and it is likely implicated as a possible mechanism involved in the initiation and progression of epileptogenesis. Flavonoids can protect the brain from oxidative stress. In the central nervous system (CNS) several flavonoids bind to the benzodiazepine site on the GABA_A_-receptor resulting in anticonvulsive effects. *Objective*. This review provides an overview about the role of flavonoids in oxidative stress in epilepsy. The mechanism of action of flavonoids and its relation to the chemical structure is also discussed. *Results/Conclusions*. There is evidence that suggests that flavonoids have potential for neuroprotection in epilepsy.

## 1. Introduction

Epilepsy is one of the oldest recorded neurological disorders, with at least 3000 years of written history [1]. It is the most common neurological condition, affecting 1-2% of the population worldwide and can profoundly affect many aspects of quality of life [2]. The incidence of epilepsy in developed countries is approximately 50 per 100,000 while that of developing countries is 100 per 100,000 affecting people of all ages, races, and social groups [3].

Epilepsy is the term used for a group of disorders characterized by recurrent spontaneous seizures and involves hyperexcitable neurons. It is assumed that there is an imbalance between inhibitory GABA-mediated and excitatory glutamate-mediated neurotransmission [4, 5]. It is commonly associated with the brain dysfunctions leading to several behavioral comorbidities [6].

The seizure activity during epilepsy decreases the antioxidant defense mechanism in the brain and increases the amount of free radicals, which further induces the oxidative stress. Free radicals (FR) can be defined as molecules or molecular fragments that contain one or more unpaired electrons [7]. These free radicals were involved in causation of lipid peroxidation, brain edema, and epilepsy, including coma and death [2].

Several antiepileptic medications are employed to manage epilepsy. However, they exhibit serious side effects, such as depression, ischemia, impaired cognition, and motor disability. Additionally, 20–30% of those afflicted have seizures that are resistant to treatment with the currently available antiepileptic drugs [2].

Traditional medicine, despite its limitations, is addressing the health care needs of millions of people worldwide. Natural products from folk remedies have significantly contributed to the discovery of modern drugs and can be an alternative source for antiepileptic drugs with novel structures and better safety and efficacy profiles [8, 9]. Phytomedicines can potentially play an important role in the development of new antiepileptic drugs [10] and may be effective in combating or preventing disease due to their antioxidant effect [11]. Phytomedicines use is very common in many parts of the world to manage various forms of epilepsies [12].

Experimental evidences have clearly demonstrated that flavonoids exert antiepileptic activity by modulating the GABA_A_-Cl-channel complex, as they are structurally similar to benzodiazepines [13]. Thus, flavonoids may have a modulating role in the treatment of neurodegenerative diseases due to their phenolic nature, since they can disrupt cellular oxidative processes in the central nervous system [14].

Several flavone derivatives may provide important leads for the development of potent and selective benzodiazepine receptor ligands. Studies on neuroactive flavonoids from herbal medicines could lead to their establishment as potential therapeutics for GABA_A_ receptor-mediated disorders [8].

This review provides an overview about the role of flavonoids on oxidative stress in epilepsy and an explanation of the main aspects involved in the development of this pathology.

## 2. Oxidative Stress

Oxidative stress is both the cause and the consequence of impaired functional homeostasis that characterizes human aging [15]. The oxidative stress is the most prominent mechanism in the development and progression of epilepsy and other diseases, including Alzheimer's disease, chronic degenerative diseases, stroke, rheumatoid arthritis, diabetes, and cancer [2].

Reactive oxygen species (ROS) are spontaneously generated in cells during metabolism and are capable of causing oxidative damage when produced in excessive quantities [16]. ROS have been implicated in the pathogenesis of a wide variety of human diseases [17] that alter the function of genetic apparatus and can oxidize and damage nucleic acids, proteins, and lipids, which are the major components of cell membranes [15], and also lead to cell death [18].

Oxidative stress is caused by excessive production of reactive oxygen (ROS) species such as hydroxyl radical (HO^•^), superoxide anion radical (O_2_^•^), hydrogen peroxide (H_2_O_2_), peroxyl radicals (HOO^•^), and high amounts of nitric oxide (NO^•^) and its derivative reactive nitrogen species (RNS) [2].

The brain is particularly prone to damage by reactive oxygen species (ROS) and reactive nitrogen species (RNS) because of its high oxygen requirement, high amount of mitochondria, and high metabolic rate. Moreover, persistent seizures promote the increase of ROS/RNS production, which cause oxidative damage of biomolecules [5]. The biochemical process by which energy released by oxidation of nutrients is converted into adenosine triphosphate by mitochondria (oxidative phosphorylation) is the most important factor for production of free radicals, in addition to the external environmental sources, such as radiation, ultraviolet light, and pollution or as bioproducts of endogenous enzymatic activities, such as in inflammatory process [15].

The physiological production of ROS in aerobic organisms requires the presence of a defense system against effects of these oxidative species. A poor defense system allows the formation of superoxide anions and hydrogen peroxide [7]. Cells possess antioxidant systems to protect themselves against dangerous ROS and antioxidants must be continually present at their sites of production at high concentrations [17].

Superoxide dismutase (SOD), catalase (CAT), and glutathione (GSH) are the most important natural antioxidants that play a role in scavenging these free radicals. Catalase is an enzyme found mainly in mitochondria and peroxisomes that catalyzes the decomposition of hydrogen peroxide to water and oxygen [19].

Catalase and hydroperoxidase enzymes convert hydrogen peroxide and hydroperoxides to nonradical forms and function as natural antioxidants in human body [18]. The hydroperoxides are a class of compounds produced as a result of phospholipid peroxidation. Its high concentration in tissues suggests that the hippocampal cells are more vulnerable to damage during the acute period of seizures [20, 21].

The superoxide radical can react with NO, generating a highly reactive molecule, the peroxynitrite anion (ONOO^−^), which is able to induce lipid oxidation and inactivate several key sulfhydryl-bearing enzymes, depleting the sulfhydryl protein content [19]. Peroxynitrite, a highly reactive radical produced by the reaction between NO and superoxide anion, leads to cytotoxicity, neurodegeneration, and apoptotic cell [15].

The SOD enzyme plays a key role in detoxifying superoxide anions in hydrogen peroxide and oxygen. It can scavenge the superoxide anion by catalyzing it to H_2_O_2_ and O_2_ which prevents the oxidative stress-induced cellular damage [22]. SOD has two isoforms, one constitutive and the other inducible. Mn-SOD (MnSOD) resides in the mitochondria and is inducible by cytokines through the pathway of NF-*κ*B and other cofactors. Copper-zinc-SOD is constitutive in eukaryotes and is mainly located in the cytoplasm but also in the lysosomes, nucleus, and spaces between membrane internal and external mitochondria and peroxisomes [23]. Deficits in SOD and catalase activities precede seizures, whereas after seizures these activities are abnormally low [24]. Superoxide dismutase is an enzyme that is found in the cytoplasm containing Cu and Zn in the active site to form a bridge with imidazole or histidine. SOD catalyzes the conversion of O_2_^•−^ to H_2_O_2_ [7].

Another molecule involved in this process is the nitric oxide, which is a molecule that is associated with regulation of neuronal stimulation ability and epileptic activity. Nitric oxide reacts with various biological molecules and induces oxidizing product, neuronal toxicity, and epileptic attacks [22]. Substances with NO-scavenging action compete with oxygen, leading to reduced production of nitrite, showing the antioxidant activity [14].

Glutathione (GSH) is considered the most prevalent and important intracellular nonprotein thiol [16]. It is an endogenous antioxidant which plays a vital role as a free-radical scavenger that protects cells against oxidative damage [25]. Modification of intracellular levels of GSH has also been shown to regulate seizure susceptibility and neuronal survival [21].

Glutathione peroxidase (GPx) is an enzyme that catalyzes the reduction of two peroxide molecules using reduced glutathione (GSH). The products of the reaction are oxidized glutathione (GSSG) and water [7]. The conversion of H_2_O_2_ to H_2_O and O_2_ is made by catalase and glutathione peroxidase.

Peroxidation of membrane polyunsaturated fatty acids produces toxic malondialdehyde (MDA), which compromises membrane lipid, matrix dynamics, and results in the progression of seizures [22]. A significant increase in MDA levels and a decrease in GSH levels indicate antioxidant-oxidant imbalance [26].

Free-radical generation can induce seizure activity by direct inactivation of glutamine synthase, thereby permitting an abnormal build-up of excitatory neurotransmitter glutamic acid. The onset of oxygen-induced convulsions in animals is correlated with a decrease in the brain cortex content of neurotransmitter GABA because of the inhibition of enzyme glutamate decarboxylase by oxygen free radicals [27].

Antioxidants play a major role in protection against molecular oxidative damage and may have therapeutical relevance in neurodegenerative diseases [28]. Plant-based foods are a budding source of natural antioxidants (ascorbic acid, *α*-tocopherol, carotenoids, flavonoid, and phenolic acids) which possess a variety of antioxidants [16]. As a result, medicinal plants recently have been given particular attention as a protective agent against epilepsy and oxidative stress [25].

## 3. Seizures

Seizures result in a remodelling of membrane phospholipids through the activation of the phospholipid degradative enzymes, phospholipases C (PLC) and A_2_/A_1_ (PLA_2_/A_1_), causing an accumulation of free fatty acids and diacylglycerol [29].

Seizures are classified as partial or generalized. Partial seizures are the result of an abnormal electrical discharge in a specific part of the brain (seizure focus). These can be simple (no change in consciousness) or complex (with decrease or loss of consciousness). Complex partial seizures originate preferentially in temporal lobe structures. They become widespread when abnormal electric activity spreads to the entire brain [7].

Glutamate and GABA are quantitatively the most important excitatory and inhibitory neurotransmitters, respectively, in the mammalian brain. Evidences suggest that alterations in these neurotransmitter systems may be associated with epilepsy [30]. Excessive stimulation of NMDA receptors, a deficiency of GABA_A_ neurons, has been proposed to underlie seizures. Thus, receptors for these two neurotransmitters are regarded as important targets for antiepileptic drugs [31, 32].

Seizures are controlled in nearly 70% of patients with epilepsy, mostly by pharmacologically modulating membrane ion channels or GABAergic or glutamatergic transmission. *γ*-Aminobutyric acid type A (GABA_A_) receptors are targets for neuroactive drugs, such as benzodiazepines, and this interaction mediates their anxiolytic, hypnotic, and anticonvulsant effects at the benzodiazepine site on the receptors [8]. Low catecholamine may be a causative factor of epilepsy [33].

The role of cholinergic neurotransmission and acetylcholinesterase (AChE) in epilepsy is well known. Acetylcholine (ACh) is a chemical mediator and important regulator of cortex and hippocampus function. It has an inherent role in the seizure cascade [26].

The selection of an antiepileptic drug for treatment is predicated on its efficacy for the specific type of seizures, tolerability, and safety [34]. Though a large number of newer antiepileptic drugs (AEDs) are available, treatment is still not satisfactory [26].

There has been considerable success in the development of novel antiepileptic drugs (AED) along with new improved formulations. These include older first generation drugs such as carbamazepine, phenobarbital, and valproic acid and newer second generation drugs such as lamotrigine, vigabatrin, tiagabine, topiramate, gabapentin, and levetiracetam [34].

Phenobarbital and diazepam, standard antiepileptic drugs, produce their antiepileptic effects by enhancing GABA neurotransmission in the brain. Phenytoin, another standard antiepileptic drug, mainly produces its antiepileptic effect by blocking sodium channels which reduces cell excitability [35]. The reduction of T-type Ca^2+^ currents by drugs such as ethosuximide can prevent seizures induced by pentylenetetrazole [36].

It has been reported that the molecular targets of some antiepileptic drugs are voltage-dependent ionic channels, including sodium, calcium, and the potassium channels and that cerebral sodium channels are modulated by some anticonvulsants agents [4].

Excessive glutamate overstimulates* N*-methyl-*D*-aspartic acid (NMDA) receptors, leading to the increase of intracellular Ca^2+^ and excitotoxicity. Generally, currently available antiepileptic drugs exert their effect by antagonizing Na^+^ or Ca^2+^ channels, facilitating GABAergic inhibition or reducing glutamatergic neurotransmission [30].

Because different models could simulate dissimilar kinds of seizures or epilepsy, selecting the appropriate model is a key factor in AED screening, in the case of false-positive or false-negative results. Prediction of a drug's efficacy on epilepsy cannot rely on one chronic model, but rather a battery of models should be used [9]. The screening of antiepilepsy drugs is based mainly on two types of models, that is, acute seizure model and chronic seizure model. Maximal electroshock test (MEST) and pentylenetetrazole (PTZ) injection model are the most commonly used acute seizure models whereas the kindling model is considered as chronic model of epilepsy that is primarily used for evaluating the test drug for antiepileptogenic activity [37].

## 4. Animal Models Used to Assess Anticonvulsant Activity

### 4.1. Maximal Electroshock Seizure (MES)

Maximal electroshock seizure test is a predictive animal tool to study anticonvulsant drug efficacy and has been documented as a major end point used to assess the antiepileptic drugs (AEDs) effective potentials against generalized tonic-clonic seizures [38]. This model has been shown to be adjusted in the reproduction of the ictal phenomenon of the focal epilepsy [4].

MES-induced convulsion model causes facilitation of Ca^2+^ and another positive ion like Na^+^ into the cells, and the blockade of them can prevent MES-induced tonic extension. Potentiation of GABA receptor may offer protection against MES-induced seizures [39]. Seizures are induced in rats by delivering electroshock of 150 mA for 0.2 s by means of a convulsiometer through a pair of ear clip electrodes. All established antiepileptic drugs have anticonvulsant activity in at least maximal electroshock seizure (MES) model [34].

MES-induced seizure can be prevented either by drugs that inhibit voltage-dependent Na^+^ channels such as phenytoin, valproate, felbamate, and lamotrigine or by drugs that block glutamatergic receptor such as felbamate [40]. Many neurotransmitter systems other than GABA, such as norepinephrine, dopamine, and serotonin (5-HT), are related to the occurrence of electroshock seizure [41].

### 4.2. Pentylenetetrazole (PTZ)

Pentylenetetrazole, a GABA_A_ receptor antagonist, is a well-established animal model to assess efficacy of drugs against generalized absence seizures in humans [42]. Seizures induced by PTZ lead to an increase in lipid, protein damage, and NO content and to a decrease in SOD and CAT activities and sulfhydryl protein content in the liver and serum of rats [19].

Pentylenetetrazole-induced seizure activity mimics the increased oxidative stress in the brain by altering membrane phospholipid metabolism and ultimately resulting in the release of free radicals. To assess the seizure activity, duration of hind limb extension is measured [2].

PTZ is known to block the action of GABA in the CNS, inducing convulsion [37], and is the most popular chemoconvulsant used for evaluating antiepileptic drugs [42]. This test represents a valid model for human generalized myoclonic and also absence seizures [36, 38].

PTZ causes epilepsy through the activation of glutamate receptors (NMDA) and stimulation of the calcium ions for entering into the nerve cells [22]. The activation of* N*-methyl-*D*-aspartate receptor appears to be involved in the initiation and generalization of the PTZ induced seizures. Accordingly, drugs that block glutamatergic excitation mediated by NMDA receptor such as felbamate have anticonvulsant activity against PTZ-induced seizures [36]. Diazepam is an effective and widely used drug for absence seizures due to GABAergic facilitating property. The involvement of NMDA receptor activation has shown to mediate the PTZ seizures [13].

### 4.3. Pilocarpine

Pilocarpine is a cholinergic agonist. Pilocarpine model of epilepsy (PME) reproduces the main features of human temporal lobe epilepsy and it is suitable to study partial epilepsy [5]. This led to cholinomimetic syndromes characterized by salivation, orofacial movement, brightening of the eyes and lacrimation, raised body furs, increased gastric motility, and urinary incontinence as well as tonic-clonic seizures of the hind limbs which mimicked the features of temporal lobe epilepsy in human beings.

Pilocarpine-induced seizures caused prominent neuronal loss in the hippocampus [21]. Pilocarpine-induced increased oxidative stress could be reflected in direct activation of some antioxidant enzymes [43]. This model can be used as animal preparations to understand the basic mechanisms of epileptogenesis [5].

### 4.4. Picrotoxin (PIC)

Picrotoxin elicits seizures by antagonizing the effect of GABA by blocking the chloride channels linked to GABA_A_-receptors. This prevents the conductance of chloride ions into the brain, thus inhibiting GABA-mediated inhibition and GABA neurotransmission [10].

### 4.5. Chemical Kindling Model

Kindling model has been widely studied both as a tool for understanding chronic epileptogenesis and as a model for testing AEDs with a potential of treating complex partial seizures. It is characterized by the development of persistent reduction in seizures threshold after a repeated administration of subconvulsant doses of stimulant drugs, such as cocaine, carbamylcholine, and pentylenetetrazole [38].

Kindling is a form of experimental epilepsy in which a periodic electrical stimulation of brain pathway induces a permanently hyperexcitable state. Stimuli trains of various intensities, durations, and frequencies are given every 60 min, 5 times a day for 5–8 consecutive days, stereotactically implanted in the Ca_3_ region of the hippocampus. Before and after electrostimulation of hippocampus bioelectrical activity is registered by electroencephalography method (EEG) using bipolar disposition [44].

### 4.6. Bicuculline

Bicuculline, a potent and selective GABA_A_ receptor antagonist, is thought to produce convulsion by blocking GABA_A_ receptors to attenuate GABA-mediated inhibition. Bicuculline produced tonic convulsions in mice [35].

### 4.7. Kainic Acid (KA)

The kainic acid seizure model is particularly useful for the study of the evolution, propagation, and pathological consequences of epileptic discharge in the limbic system. KA increases ROS production, mitochondrial dysfunction, and apoptosis in neurons in many regions of the brain, particularly in the hippocampal regions [43].

## 5. Involvement of GABA in Epilepsy

GABA is the most important inhibitory neurotransmitter in the human central nervous system. The GABA receptors are widely distributed in mammalian brain and are the target for antiepileptic drugs [5]. GABA_A_ receptors are heteromeric GABA-gated chloride channels. The transmembrane ion channel is opened by a stimulus generated by GABA, which allows an influx of chloride ions. This results in a decrease of the depolarizing effects of an excitatory input, thereby depressing excitability [1].

In addition to GABA binding sites, the GABA_A_ receptor possesses binding sites for compounds that allosterically modify the chloride channel gating of GABA, such as benzodiazepines and barbiturates [5]. An increase in the inhibition transmission, particularly mediated by GABA, could lead to sedative and anticonvulsant effects [45].

Ligands for the benzodiazepine binding site modulate the inhibitory effects of GABA. Such ligand binding sites are classified as positive allosteric modulators, antagonists, or negative allosteric modulators according to their spectrum of intrinsic efficacy towards the GABA_A_ receptor [46]. Positive allosteric modulators increase the frequency of chloride channel openings without altering channel conductance or duration of opening [41].

Benzodiazepines (BDZs) site agonists increase the GABA-induced chloride channel opening frequency and have established efficacy in the treatment of anxiety, insomnia and epilepsy, muscle relaxant, sedative hypnotic, and cognition-impairing effects [47].

There is compelling evidence of an imbalance between excitatory and inhibitory neurotransmitters in the pathophysiology of epilepsy. Decrease in GABAergic and glutamatergic transmission is involved in the generation of epilepsy [1].

The search for new GABA_A_ receptor ligands has led to the identification of a variety of high-affinity compounds structurally different from the BDZ nucleus, such as *β*-carbolines, trizolopyridazines, quinolines, and flavonoids [47]. When a compound binds to the GABA-benzodiazepine site it can lead to either anticonvulsive activity or a sedative effect, depending on the receptor subtype the compound is binding to [1].

Current research has found that antioxidants may provide neuroprotection, to a certain degree, against the neurotoxicity of seizures at the cellular level [21]. Flavonoids are compounds that exhibit, among other activities, an action recognized for its antioxidant properties. Thus, the objective of this work is to discuss the role of flavonoids on oxidative stress in epilepsy.

## 6. Flavonoids with Action in the CNS

A number of ongoing scientific studies are isolating and characterizing the bioactive principles of the medicinal plants which can serve as “lead” compounds in drug development processes. It is a fact that the vegetal kingdom has been found to be a rich source of bioactive compounds [48], which are considered members of a specific family of phytochemicals that can interact with multiple targets [49].

The metabolites possess various biological activities, including anti-inflammatory, cytostatic, antiviral properties and antinociceptive activity. Furthermore, it is known that some flavonoids, as well as their glycosides, exert anxiolytic, sedative, and anticonvulsant effects on the central nervous systems (CNS) [50].

The phenylpropanoids derived from caffeic acid exhibit neuroprotective effects [45]. Polyphenolics, the group of antioxidants containing a polyphenolic substructure, include flavonoid and nonflavonoid polyphenolics [43]. Recently polyphenolics have been given considerable scientific and therapeutic interest because they offer protection from free radicals damage [2].

The flavonoids are present in fruits and vegetables of plants nuts, plant-derived beverages, traditional eastern medicines, and herb-containing dietary supplements. They are known to be antioxidants that can protect the cell from oxidative stress [42]. The bioactivities of flavonoids are largely dependent on the structure and exhibit a wide range of biological activities [51].

More than 5000 different flavonoids have been isolated, and the pharmacological properties of many of them have been described [52]. The flavonoids from the medicinal plants such as valerian (*Valeriana officinalis*), chamomile (*Matricaria recutita*), and kava-kava (*Piper methysticum*) have sedative-hypnotic effects based on positive allosteric modulation of GABA_A_ receptors [33].

The flavonoids belonging to these different chemical classes inhibit, at varying degrees, enzymes that phosphorylate (kinases) and dephosphorylate (phosphatases) critical proteins that signal transduction pathways, which regulate oxidative stress, inflammation, and cell survival [53]. These metabolites have been reported to have anticonvulsant activity [49]. It has been previously shown in several studies that rutin, quercetin, and isoquercitrin have anticonvulsant effects on experimental epilepsy models [10]. Experimental evidences clearly demonstrated that flavonoids exert antiepileptic activity by modulating the GABA_A_-Cl-channel complex, as they are structurally similar to benzodiazepines [13].

The work by Gupta et al. [3] showed that morusin was able to ameliorate the epileptic seizures induced by MES, exhibited significant activity in MES-induced seizure models, and can be an effective compound against both grand mal and petit mal epilepsies.

In another study to evaluate the activity of ethanol extract of the* Abelmoschus manihot* the PTZ induced convulsion model was used. The extract showed anticonvulsant effect after oral administration. The potential active components of extract* in vivo* were identified as isoquercitrin, hyperoside, hibifolin, quercetin-3-*O*-glucoside, quercetin, and isorhamnetin [37].

The phytochemical investigations of* Waltheria indica* L. (syn.* Waltheria americana*) showed that the flavonoids (−)-epicatechin, quercetin, kaempferol, and kaempferol-3-*O*-*β*-*D*-(6′′-*E*-*p*-coumaroyl)-glucopyranoside possess therapeutic potential in the prevention of oxidative stress. The hexane extract from leaves of* Waltheria indica* exhibited higher antioxidant activity than ascorbic acid and *α*-tocopherol [54].

Phytochemical research carried out with* Passiflora incarnata* had led to the isolation of several bioactive metabolites like chrysin, apigenin, homoorientin, vitexin, luteolin, quercetin, kaempferol, isovitexin, orientin, isoorientin, schaftoside, isoschaftoside, harman, harmol, harmine, harmalol, and harmaline. Several CNS depressant effects of* Passiflora incarnata* have been experimentally explored and suggested principally due to the presence of flavonoids like chrysin to act by agonizing the benzodiazepine receptor [6].

Chrysin (5,7-dihydroxyflavone), a flavonoid, was found to be a ligand for benzodiazepine receptors. This flavonoid, assayed* in vitro*, had moderately high affinity for the central BDZ-receptors and displayed anticonvulsant properties* in vivo*. Tonic-clonic seizures induced by PTZ were prevented by intracerebroventricular injection of chrysin. A central benzodiazepine receptor antagonist, flumazenil, abolished this effect [55].

Apigenin (5,7,4′-trihydroxyflavone) is one of the most common flavones. The presence of apigenin has been detected previously in* Tanacetum parthenium*. This flavone has been characterized as a centrally acting benzodiazepine ligand which shows CNS effects after systemic administration. In the model of picrotoxin-induced convulsions in rats, the apigenin was able to reduce the latency of onset and locomotor activity [56].

The flavonoids and isoflavonoids are probably electron donors. They have B-ring conjugated chemical structures rich in hydroxyl groups, which have potential antioxidant actions by reacting with and inactivating superoxide anions, oxygen lipid peroxide radicals, and/or stabilizing free radicals involved in the oxidative process by hydrogenation or complexing with oxidant species [14].

Therefore, OH^•^ removal by apigenin displayed a considerable antioxidant activity and may be capable of inhibiting cell damage caused by that radical and significantly decreasing the production of nitrite [14], acting as CNS-active molecules, in other words, as partial agonists of the GABA_A_ receptor [52]. Apigenin also may block glutamatergic transmission through kainic acid receptors to prevent the generation and propagation of seizure-related neuronal discharges, potentially through inhibitory systems in the central nervous system. This flavonoid also has considerable antiexcitotoxicity effects [57].

Vitexin (5,7,4-trihydroxyflavone-8-glucoside) is a* C*-glycosylated flavone found in a number of plants such as* Passiflora* sp., bamboo leaves. It has several pharmacological properties, exerting its anticonvulsant effects by increasing the seizure-onset time in PTZ-induced minimal clonic seizures (MCS) and generalized tonic-clonic seizures (GTCS), probably through binding at the benzodiazepine site of the GABA_A_ receptor complex [58].

Luteolin (3′,4′,5,7-tetrahydroxyflavone), a flavone contained in chamomile and chrysanthemum, has potent antioxidant, neuroprotective, anxiolytic, and anti-inflammatory properties [59, 60] and decreased iNOS levels in PTZ induced seizures. Antioxidant effect of luteolin has not been revealed directly, but the increase of eNOS in brain and other tissues and decrease of iNOS show that luteolin has an indirect antioxidant effect [61].

Quercetin (3,3′,4′,5,7-pentahydroxyflavone) is a flavonoid found in a variety of fruits and vegetables. The protective and toxic effects of quercetin depend on both timing and dose with regard to the anticonvulsant effects, as shown in the study performed by Nassiri-Asl et al. [42]. It is possible that quercetin modulates GABA_A_ receptors and also acts as NMDA receptor antagonist [13].

Four flavonoids were extracted from* Scutellaria baicalensis* (baicalein, scutellarein, wogonin, and baicalein). Wogonin possesses central nervous system effects. It increased Cl^−^ influx into the intracellular area and produced the anticonvulsive effects mediated by the GABAergic neuron. The use of flumazenil inhibited the Cl^−^ influx induced by wogonin. These results indicate that wogonin may be a partial agonist for GABA_A_ receptors and this property may contribute to its anticonvulsant effect as well as the highest affinity for benzodiazepine site [41].

A number of flavonoids including wogonin, oroxylin A, and baicalin have been isolated from a traditional Chinese herb Huangqin, the dry root of* Scutellaria baicalensis* Georgi. The constituent baicalin has been found to interact with the BDZ site of GABA_A_ receptors, a partial agonist exhibiting no amnesic, anticonvulsant, or motor incoordination activities in mice [62].

Rutin (3′,4′,5,7-tetrahydrohyflavone-3-rhamnoglucoside), a flavonoid of the flavonol type, is found in many plants such as buckwheat, apples, and black tea. It has several noteworthy pharmacological properties, including antioxidant, anticarcinogenic, cytoprotective, antiplatelet, antithrombotic, vasoprotective, cardioprotective, and neuroprotective activities [63]. The central administration of rutin dose-dependently affects epileptic seizures induced by PTZ. It has ameliorated ischemia-reperfusion injury in the brain and may enhance memory retrieval in normal animals. These effects could potentially be related to the antioxidative effects of rutin. It is possible that rutin is a ligand for benzodiazepine receptors through positive allosteric modulation of the GABA_A_ receptor [8].

Hesperidin is a naturally occurring flavanone that occurs in citrus and other plants. This glycoside is deglycosylated partly in the gut by bacteria to give hesperetin, its so-called aglycone. Both compounds were able to block the effects of enhanced calcium. There is possibility for use of both compounds to control pathophysiological disturbances of brain excitability in drug abuse and epilepsy [64].

Epigallocatechin-3-gallate (EGCG), the main polyphenol of green tea, has been characterized as having antioxidant. It has been found to be an effective antioxidant and could reduce oxidative brain damage after PTZ-induced kindling. The protective effect of EGCG against PTZ-induced kindling could be partly attributed to its potential antioxidant effect [25].

Hispidulin is a naturally occurring flavone commonly found in* Saussurea involucrata*. Hispidulin has been confirmed to penetrate the blood-brain barrier (BBB). It possesses antiepileptic activity and contributes to the decrease in glutamate release, not only by attenuating voltage-dependent Ca^2+^ entry but also by directly interfering with the exocytotic machinery release itself [30].

Linarin, a main flavone glycoside of* Chrysanthemum boreale*, was one of the active principles of the plant and may prevent CNS excitation or stress, since it has been shown to inhibit PTZ-induced convulsion. Linarin and its aglycone, acacetin, exhibited sedative and anticonvulsant activities* in vivo* assays. This aglycone was also active in pentobarbital-induced assay and PTZ-induced assay in mice and showed more potent activities than its glycoside, linarin [65].

Flavonoids have been shown to influence peripheral blood flow in humans. For example, in a brain imaging study, the consumption of flavanol-rich cocoa enhanced cortical blood flow. This result is important when considering mechanisms that increase cerebrovascular function, especially in the hippocampus, a brain region that is important for memory and that may facilitate adult neurogenesis [63].

Recently,* Baikal skullcap* and its major flavonoids (baicalin, baicalein, and wogonin) have attracted attention for their diverse activities, including anti-inflammatory, antioxidative, anticonvulsant, and anxiolytic activities. These flavonoids have been previously shown to have high affinity for the benzodiazepine binding site of GABA_A_ receptor [66].

Baicalin can pass through blood-brain barrier, may be a potential supplement in the prevention and treatment of epilepsy, decreased the percentage of seizures, increased the latency to the onset of seizure, inhibited the increase in lipid peroxidation level, NO content, and the decrease of GSH activities, and exerted neuroprotective effects [21].

Various synthetic derivatives of flavonoids have been studied. Among these, much work has focused on 6-bromoflavone and 6-bromo-3′-nitroflavone, which were found to display specific affinity for BDZ-receptors. These two synthetic flavonoids also showed anxiolytic-like effects in the mouse elevated plus-maze [46].

*Ginkgo biloba* leaves contain a number of flavonoids (kaempferol, quercetin, and isorhamnetin derivatives) [29]. The biflavonoid amentoflavone is found in a number of plants with medicinal properties, including* Ginkgo biloba* and* Hypericum perforatum*. It has been shown to exhibit high affinity to brain benzodiazepine receptors in vitro. As a result, the cell is inhibited and an anticonvulsant activity is achieved [1].

The naringenin is a flavanone that was isolated from the ethanolic leaf extract of* Mentha aquatic* and had high activity to the GABA-benzodiazepine receptor. This flavanone has been shown to pass the blood-brain barrier, which means that it can exercise an effect on the CNS [56]. However, in the work of Shrestha et al. [33], the naringenin, one of the monomers of rhusflavone, has only weak GABA_A_-BZD receptor binding.

In the aerial parts of* Hypericum perforatum* numerous polyphenolic constituents are present mainly belonging to the flavonoids, proanthocyanidins, biflavonoids, and flavones and flavonols: quercitrin, hyperin, rutin, and amentoflavone. Some previous investigations have shown that tannins from* Hypericum perforatum* have anticonvulsive effect. Thanks to their well-known antioxidant ability, these compounds may have a protective antiepileptic role too [44].

Goodyerin is a flavonol glycoside isolated from the whole plants of* Goodyera schlechtendaliana,* which has significant sedative and anticonvulsant effects on the CNS. When compared, the structures of goodyerin and rutin show only a 4-hydroxy-3,5-dimethoxyphenyl group added in the former. The reason by which goodyerin strongly inhibits the CNS is still unknown [52].

Fisetin is a tetrahydroxy flavonoid which contains hydroxyl groups on the basic C_6_-C_3_-C_6_ skeleton. Fisetin reduced both electrical kindling seizures in rats and acute seizures in PTZ, strychnine, isoniazid, or MES tests in mice. The anticonvulsant effect may be ascribed to protection of endogenous enzyme level, increase of the GABA level in brain, and inhibition of oxidative injury [9].

6-Hydroxyflavone (6HF) is a naturally occurring flavone found in the leaves of* Barleria prionitis*, a species of plants in the Acanthaceae family native to India with bind to GABA_A_ receptors benzodiazepine site with moderate binding affinity. 6HF is a partial agonist of GABA_A_ receptors [47].

Several flavone derivatives may provide important leads for developing potent and selective benzodiazepine receptor ligands as potential therapeutic agents for GABA_A_ receptor-mediated disorders [58].

## 7. Conclusions and Future Directions

Increased production of free radicals leads to the development of several pathologies like epilepsy, which is among the three most common neurological disorders and involves significant social impact. The development of seizures is accompanied by the increased formation of reactive oxygen species, an important pathogenic factor, resulting in oxidative stress involved neuronal death.

An important factor in the pharmacological therapy of this disease is that only 30% of patients do not respond to the therapy. Among the reasons, there are the long-term use and side effects like the hepatotoxicity [61].

Due to the side effects of drugs used for treatment of epilepsy, it is important and necessary to search for new therapeutic options that cause a reduction of these undesirable effects.

The flavonoids play a significant role in this regard by its antioxidant capacity. Many studies show that flavonoids act on GABA receptor potentiating its effect. So, it is a promising metabolite that may act in CNS disorders, including epilepsy.

## References

[1] Svenningsen A. B., Madsen K. D., Liljefors T., Stafford G. I., Staden J. V., Jäger A. K. (2006). Biflavones from *Rhus* species with affinity for the GABA_A_/ benzodiazepine receptor. *Journal of Ethnopharmacology*.

[2] Ramalingam R., Nath A. R., Madhavi B. B., Nagulu M., Balasubramaniam A. (2013). Free radical scavenging and antiepileptic activity of *Leucas lanata*. *Journal of Pharmacy Research*.

[3] Gupta G., Dua K., Kazmi I., Anwar F. (2014). Anticonvulsant activity of Morusin isolated from *Morus alba*: modulation of GABA receptor. *Biomedicine & Aging Pathology*.

[4] de Almeida R. N., de Sousa D. P., Nóbrega F. F. D. F. (2008). Anticonvulsant effect of a natural compound *α*,*β*-epoxy-carvone and its action on the nerve excitability. *Neuroscience Letters*.

[5] Grosso C., Valentão P., Ferreres F., Andrade P. B. (2013). The use of flavonoids in central nervous system disorders. *Current Medicinal Chemistry*.

[6] Singh B., Singh D., Goel R. K. (2012). Dual protective effect of *Passiflora incarnata* in epilepsy and associated post-ictal depression. *Journal of Ethnopharmacology*.

[7] Cardenas-Rodriguez N., Huerta-Gertrudis B., Rivera-Espinosa L. (2013). Role of oxidative stress in refractory epilepsy: evidence in patients and experimental models. *International Journal of Molecular Sciences*.

[8] Nassiri-Asl M., Shariati-Rad S., Zamansoltani F. (2008). Anticonvulsive effects of intracerebroventricular administration of rutin in rats. *Progress in Neuro-Psychopharmacology & Biological Psychiatry*.

[9] Zhu H.-L., Wan J.-B., Wang Y.-T. (2014). Medicinal compounds with antiepileptic/anticonvulsant activities. *Epilepsia*.

[10] Orhan N., Orhan D. D., Aslan M., Ükürolu M., Orhan I. E. (2012). UPLC-TOF-MS analysis of *Galium spurium* towards its neuroprotective and anticonvulsant activities. *Journal of Ethnopharmacology*.

[11] Akinmoladun A. C., Ibukun E. O., Afor E., Obuotor E. M., Farombi E. O. (2007). Phytochemical constituent and antioxidant activity of extract from the leaves of *Ocimum gratissimum*. *Scientific Research and Essay*.

[12] Okoye T. C., Akah P. A., Omeke C. P. (2010). Evaluation of the anticonvulsant and muscle relaxant effects of the methanol root bark extracts of *Annona senegalensis*. *Asian Pacific Journal of Tropical Medicine*.

[13] Choudhary N., Bijjem K. R. V., Kalia A. N. (2011). Antiepileptic potential of flavonoids fraction from the leaves of *Anisomeles malabarica*. *Journal of Ethnopharmacology*.

[14] Marques T. H. C., de Melo C. H. S., de Carvalho R. B. F. (2013). Phytochemical profile and qualification of biological activity of an isolated fraction of *Bellis perennis*. *Biological Research*.

[15] Dato S., Crocco P., D'Aquila P. (2013). Exploring the role of genetic variability and lifestyle in oxidative stress response for healthy aging and longevity. *International Journal of Molecular Sciences*.

[16] Aseervatham G. S. B., Sivasudha T., Sasikumar J. M., Christabel P. H., Jeyadevi R., Ananth D. A. (2014). Antioxidant and hepatoprotective potential of *Pouteria campechiana* on acetaminophen-induced hepatic toxicity in rats. *Journal of Physiology and Biochemistry*.

[17] Jeong J.-M., Choi C.-H., Kang S.-K., Lee I.-H., Lee J.-Y., Jung H. (2007). Antioxidant and chemosensitizing effects of flavonoids with hydroxy and/or methoxy groups and structure-activity relationship. *Journal of Pharmacy & Pharmaceutical Sciences*.

[18] Pourmorad F., Hosseinimehr S. J., Shahabimajd N. (2006). Antioxidant activity, phenol and flavonoid contents of some selected Iranian medicinal plants. *African Journal of Biotechnology*.

[19] Rodrigues A. D., Scheffel T. B., Scola G. (2013). Purple grape juices prevent pentylenetetrazol-induced oxidative damage in the liver and serum of Wistar rats. *Nutrition Research*.

[20] Freitas R. M. (2009). Investigation of oxidative stress involvement in hippocampus in epilepsy model induced by pilocarpine. *Neuroscience Letters*.

[21] Liu Y.-F., Gao F., Li X.-W. (2012). The anticonvulsant and neuroprotective effects of Baicalin on pilocarpine-induced epileptic model in rats. *Neurochemical Research*.

[22] Kiasalari Z., Khalili M., Roghani M., Heidari H., Azizi Y. (2013). Antiepileptic and antioxidant effect of hydroalcoholic extract of *Ferula assa* foetida gum on pentylenetetrazole induced kindling in male mice. *Basic and Clinical Neuroscience*.

[23] Halliwell B., Gutteridge J. (2007). *Free Radicals in Biology and Medicine*.

[24] da Costa J. S., de Almeida A. A. C., Tomé A. D. R., Citó A. M. D. G. L., Saffi J., de Freitas R. M. (2011). Evaluation of possible antioxidant and anticonvulsant effects of the ethyl acetate fraction from *Platonia insignis* Mart. (Bacuri) on epilepsy models. *Epilepsy & Behavior*.

[25] Xie T., Wang W.-P., Mao Z.-F. (2012). Effects of epigallocatechin-3-gallate on pentylenetetrazole-induced kindling, cognitive impairment and oxidative stress in rats. *Neuroscience Letters*.

[26] Pahuja M., Mehla J., Reeta K. H., Joshi S., Gupta Y. K. (2011). Hydroalcoholic extract of Zizyphus jujuba ameliorates seizures, oxidative stress, and cognitive impairment in experimental models of epilepsy in rats. *Epilepsy & Behavior*.

[27] Naziroğlu M., Akay M. B., Çelik Ö., Yildirim M. I., Balci E., Yürekli V. A. (2013). *Capparis ovata* modulates brain oxidative toxicity and epileptic seizures in pentylentetrazol-induced epileptic rats. *Neurochemical Research*.

[28] Russo A., Borrelli F., Campisi A., Acquaviva R., Raciti G., Vanella A. (2003). Nitric oxide-related toxicity in cultured astrocytes: effect of *Bacopa monniera*. *Life Sciences*.

[29] Smith P. F., Maclennan K., Darlington C. L. (1996). The neuroprotective properties of the *Ginkgo biloba* leaf: a review of the possible relationship to platelet-activating factor (PAF). *Journal of Ethnopharmacology*.

[30] Lin T.-Y., Lu C.-W., Wang C.-C., Lu J.-F., Wang S.-J. (2012). Hispidulin inhibits the release of glutamate in rat cerebrocortical nerve terminals. *Toxicology and Applied Pharmacology*.

[31] Zapata-Sudo G., Mendes T. C. F., Kartnaller M. A. (2010). Sedative and anticonvulsant activities of methanol extract of *Dorstenia arifolia* in mice. *Journal of Ethnopharmacology*.

[32] Zhang F., Liu J., Shi J. (2010). Anti-inflammatory activities of resveratrol in the brain: role of resveratrol in microglial activation. *European Journal of Pharmacology*.

[33] Shrestha S., Park J.-H., Lee D.-Y. (2012). *Rhus parviflora* and its biflavonoid constituent, rhusflavone, induce sleep through the positive allosteric modulation of GABA_A_-benzodiazepine receptors. *Journal of Ethnopharmacology*.

[34] Mallesha L., Mohana K. N., Veeresh B. (2012). Synthesis and biological activities of Schiff bases of gabapentin with different aldehydes and ketones: a structure-activity relationship study. *Medicinal Chemistry Research*.

[35] Amabeoku G. J. (2008). Anticonvulsant activity of *Nylandtia spinosa* L. Dumont (Polygalaceae) aqueous and methanol leaf extracts in mice. *Human and Experimental Toxicology*.

[36] Khodaparast A., Sayyah M., Sardari S. (2012). Anticonvulsant activity of hydroalcoholic extract and aqueous fraction of *Ebenus stellata* in mice. *Iranian Journal of Basic Medical Sciences*.

[37] Guo J., Xue C., Duan J.-A., Qian D., Tang Y., You Y. (2011). Anticonvulsant, antidepressant-like activity of *Abelmoschus manihot* ethanol extract and its potential active components *in vivo*. *Phytomedicine*.

[38] Quintans L. J., Almeida J. R. G. S., Lima J. T. (2008). Plants with anticonvulsant properties—a review. *Brazilian Journal of Pharmacognosy*.

[39] Das S., Sarma P. (2014). A study on the anticonvulsant and antianxiety activity of ethanolic extract of *Punica granatum* Linn. *International Journal of Pharmacy and Pharmaceutical Sciences*.

[40] Khoshnood-Mansoorkhani M. J., Moein M. R., Oveisi N. (2010). Anticonvulsant activity of teucrium polium against seizure induced by PTZ and MES in mice. *Iranian Journal of Pharmaceutical Research*.

[41] Park H. G., Yoon S. Y., Choi J. Y. (2007). Anticonvulsant effect of wogonin isolated from *Scutellaria baicalensis*. *European Journal of Pharmacology*.

[42] Nassiri-Asl M., Naserpour Farivar T. N., Abbasi E. (2013). Effects of rutin on oxidative stress in mice with kainic acid-induced seizure. *Journal of Integrative Medicine*.

[43] Shin E.-J., Jeong J. H., Chung Y. H. (2011). Role of oxidative stress in epileptic seizures. *Neurochemistry International*.

[44] Ivetic V., Popovic M., Mimica-Dukic N., Barak O., Pilija V. (2002). St. John's wort (*Hypericum perforatum* L.) and kindling epilepsy in rabbit. *Phytomedicine*.

[45] Neto A. C., Netto J. C., Pereira P. S. (2009). The role of polar phytocomplexes on anticonvulsant effects of leaf extracts of *Lippia alba* (Mill.) N.E. Brown chemotypes. *Journal of Pharmacy and Pharmacology*.

[46] Griebel G., Perrault G., Tan S., Schoemaker H., Sanger D. J. (1999). Pharmacological studies on synthetic flavonoids: comparison with diazepam. *Neuropharmacology*.

[47] Ren L., Wang F., Xu Z., Chan W. M., Zhao C., Xue H. (2010). GABA_A_ receptor subtype selectivity underlying anxiolytic effect of 6-hydroxyflavone. *Biochemical Pharmacology*.

[48] Magaji M. G., Yaro A. H., Musa A. M., Anuka J. A., Abdu-Aguye I., Hussaini I. M. (2012). Central depressant activity of butanol fraction of *Securinega virosa* root bark in mice. *Journal of Ethnopharmacology*.

[49] Sucher N. J. (2006). Insights from molecular investigations of traditional Chinese herbal stroke medicines: implications for neuroprotective epilepsy therapy. *Epilepsy and Behavior*.

[50] Estrada-Reyes R., Martínez-Vázquez M., Gallegos-Solís A., Heinze G., Moreno J. (2010). Depressant effects of *Clinopodium mexicanum* Benth. Govaerts (Lamiaceae) on the central nervous system. *Journal of Ethnopharmacology*.

[51] Quintans J. S. S., Antoniolli Â. R., Almeida J. R. G. S., Santana-Filho V. J., Quintans-Júnior L. J. (2014). Natural products evaluated in neuropathic pain models—a systematic review. *Basic and Clinical Pharmacology and Toxicology*.

[52] Du X.-M., Sun N.-Y., Takizawa N., Guo Y.-T., Shoyama Y. (2002). Sedative and anticonvulsant activities of goodyerin, a flavonol glycoside from *Goodyera schlechtendaliana*. *Phytotherapy Research*.

[53] Keddy P. G. W., Dunlop K., Warford J. (2012). Neuroprotective and anti-inflammatory effects of the flavonoid-enriched fraction AF4 in a mouse model of hypoxic-ischemic brain injury. *PLoS ONE*.

[54] Zongo F., Ribuot C., Boumendjel A., Guissou I. (2013). Botany, traditional uses, phytochemistry and pharmacology of *Waltheria indica* L. (syn. *Waltheria americana*): a review. *Journal of Ethnopharmacology*.

[55] Medina J. H., Viola H., Wolfman C. (1998). Neuroactive flavonoids: new ligands for the Benzodiazepine receptors. *Phytomedicine*.

[56] Jäger A. K., Krydsfeldt K., Rasmussen H. B. (2009). Bioassay-guided isolation of apigenin with GABA-benzodiazepine activity from *Tanacetum parthenium*. *Phytotherapy Research*.

[57] Han J. Y., Ahn S. Y., Kim C. S. (2012). Protection of apigenin against kainate-induced excitotoxicity by anti-oxidative effects. *Biological and Pharmaceutical Bulletin*.

[58] Abbasi E., Nassiri-Asl M., Shafeei M., Sheikhi M. (2012). Neuroprotective effects of vitexin, a flavonoid, on pentylenetetrazole-induced seizure in rats. *Chemical Biology and Drug Design*.

[59] Shaikh M. F., Tan K. N., Borges K. (2013). Anticonvulsant screening of luteolin in four mouse seizure models. *Neuroscience Letters*.

[60] Theoharides T. C., Zhang B. (2011). Neuro-inflammation, blood-brain barrier, seizures and autism. *Journal of Neuroinflammation*.

[61] Birman H., Dar K. A., Kapucu A., Acar S., Üzüm G. (2012). Effects of luteolin on liver, kidney and brain in pentylentetrazol-induced seizures: involvement of metalloproteinases and NOS activities. *Balkan Medical Journal*.

[62] Wang F., Xu Z., Ren L., Tsang S. Y., Xue H. (2008). GABA_A_ receptor subtype selectivity underlying selective anxiolytic effect of baicalin. *Neuropharmacology*.

[63] Nassiri-Asl M., Mortazavi S.-R., Samiee-Rad F. (2010). The effects of rutin on the development of pentylenetetrazole kindling and memory retrieval in rats. *Epilepsy and Behavior*.

[64] Dimpfel W. (2006). Different anticonvulsive effects of hesperidin and its aglycone hesperetin on electrical activity in the rat hippocampus in-vitro. *Journal of Pharmacy and Pharmacology*.

[65] Nugroho A., Lim S.-C., Choi J., Park H.-J. (2013). Identification and quantification of the sedative and anticonvulsant flavone glycoside from *Chrysanthemum boreale*. *Archives of Pharmacal Research*.

[66] Zhang Z., Lian X.-Y., Li S., Stringer J. L. (2009). Characterization of chemical ingredients and anticonvulsant activity of *American skullcap* (*Scutellaria lateriflora*). *Phytomedicine*.

